# From intracellular processing to renal phenotypes: a mechanism-informed framework for interpreting kidney injury and electrolyte patterns with antibody–drug conjugates in solid tumors

**DOI:** 10.3389/fphar.2026.1824554

**Published:** 2026-06-26

**Authors:** Yuxin Jiang, Jianing Xu, Zikun Wang, Shoulin Zhang

**Affiliations:** 1 College of Traditional Chinese Medicine, Changchun University of Chinese Medicine, Changchun, China; 2 Nephropathy Department, The Affiliated Hospital to Changchun University of Chinese Medicine, Changchun, China

**Keywords:** acute kidney injury, antibody–drug conjugates, electrolyte disturbances, intracellular trafficking, nephrotoxicity, proximal tubulopathy, solid tumors

## Abstract

**Background:**

Antibody–drug conjugates (ADCs) have transformed solid-tumor therapy and are increasingly benchmarked directly against physician’s-choice chemotherapy. However, kidney adverse events and electrolyte disturbances remain inconsistently captured across trials and are often interpreted without a unified pharmacological framework.

**Objective:**

This review proposes a mechanism-informed narrative framework linking ADC architecture, intracellular processing, and payload exposure pathways to renal injury and electrolyte phenotype hypotheses while contextualizing these patterns against canonical renal-electrolyte programs of conventional cytotoxic chemotherapy.

**Approach:**

We conducted a targeted literature review prioritizing randomized or registrational trials, regulatory labels, real-world or pharmacovigilance data when informative, and phenotype-defining case reports supported by urinary indices, biopsy, or both when available. Evidence is presented using explicit source-level stratification rather than quantitative pooling.

**Key Messages:**

Intact ADCs are IgG-based macromolecules with limited glomerular filtration under an intact filtration barrier, whereas released small-molecule payloads or payload-containing catabolites may gain a filtration advantage and create proximal tubular exposure. Comparative studies support toxicity redistribution rather than uniform toxicity reduction. Enfortumab vedotin most often illustrates a systemic/prerenal acute kidney injury (AKI) axis driven by hyperglycemia, diabetic ketoacidosis, infection, and dehydration; sacituzumab govitecan combines gastrointestinal volume depletion with rare biopsy-proven acute tubulointerstitial nephritis; and trastuzumab deruxtecan has generated a hypothesis-defining Fanconi-like proximal tubule signal. Kidney tissue, especially the proximal tubule, contains protease-rich endolysosomal and apical brush-border systems that make renal processing of ADC-derived material biologically plausible, but kidney-specific proof remains limited. Because creatinine-based endpoints may miss mild tubular injury, future ADC programs should consider electrolyte-focused monitoring and the exploration of tubular injury biomarkers.

**Conclusion:**

A trafficking-aligned taxonomy may improve the interpretation of ADC-associated renal and electrolyte events. Kidney Disease: Improving Global Outcomes (KDIGO)-aligned kidney endpoints, standardized electrolyte reporting, and prospective validation of tubular injury biomarkers should be priorities for future ADC trials.

## 1 Introduction

The relationship between kidney disease and solid tumors is increasingly shaped by a rapidly evolving therapeutic landscape that includes not only conventional cytotoxic agents but also targeted biologics and antibody-based therapeutics (KDIGO, 2020). The Kidney Disease: Improving Global Outcomes (KDIGO) onco-nephrology conference report emphasized the high prevalence of chronic kidney disease (CKD) in oncology populations, the adverse prognostic implications of kidney dysfunction, and the practical challenges of estimating glomerular filtration rate (GFR) in patients with cancer-associated sarcopenia or cachexia (KDIGO, 2020). As a result, onco-nephrology increasingly requires frameworks that do more than list adverse events: they must connect pharmacology, tissue exposure, and syndrome recognition.

Conventional chemotherapy remains a useful benchmark because many agents exhibit mechanistically coherent renal and electrolyte toxicity programs. Cisplatin exemplifies transporter-mediated tubular injury with persistent magnesium wasting; high-dose methotrexate (HDMTX) exemplifies crystal nephropathy that is preventable by hydration, urine alkalinization, and rescue algorithms; and ifosfamide remains the textbook comparator for acquired Fanconi syndrome and proximal tubular dysfunction (Romani, 2015; Howard et al., 2016; Nardelli et al., 2025; Saito et al., 2017; Widemann et al., 2014; Ramsey et al., 2018; Zamlauski-Tucker et al., 1994). These programs are not merely historical comparators. They provide pattern anchors that help distinguish direct tubular toxicity, prerenal physiology, interstitial injury, and endothelial microangiopathic programs when new agents enter practice.

Antibody–drug conjugates (ADCs) are often described as “targeted chemotherapy,” but their safety cannot be inferred from tumor selectivity alone. An ADC integrates an antibody, a linker, and a cytotoxic payload, and each component shapes off-target exposure (Metrangolo and Engelholm, 2024; Nguyen et al., 2023; Tarantino et al., 2022; Aggarwal et al., 2023; Maecker et al., 2023; Jain et al., 2015). Clinically relevant toxicities may reflect not only target expression but also linker stability, lysosomal degradation, and linker-dependent intracellular processing, systemic shedding of filterable payload species, bystander diffusion, non-target uptake, and tissue-specific catabolic capacity (Metrangolo and Engelholm, 2024; Nguyen et al., 2023; Zhao et al., 2018; Tarantino et al., 2022; Ogitani et al., 2016; Giugl et al., 2022; Jain et al., 2015; Erickson et al., 2006; Doronina et al., 2003). Consequently, kidney safety in ADC programs should not be reduced to a binary question of whether ADCs are simply “less nephrotoxic” than chemotherapy.

In this review, we, therefore, propose a mechanism-informed, phenotype-oriented interpretive framework for kidney injury and electrolyte disturbances with ADCs in solid tumors. We explicitly distinguish observed clinical evidence from mechanistic inference, organize drug-specific synthesis in a trial-first manner, and reposition case reports as phenotype-defining rather than incidence-defining evidence. We also place mechanisms of renal handling earlier in the manuscript so that subsequent clinical interpretation is grounded in pharmacology and renal cell biology from the outset.

## 2 Scope, evidence identification, and terminology

### 2.1 Scope, sources, and review logic

We performed a targeted literature review designed to be transparent and reproducible without claiming a PRISMA-level systematic synthesis. The review focused on adults with solid tumors treated with ADCs that currently have mature or registrational clinical evidence in solid tumors, with conventional chemotherapy used as a mechanistic comparator when relevant. Evidence was prioritized in the following order: (i) randomized or registrational trials, (ii) drug labels and regulatory summaries, (iii) real-world cohorts or pharmacovigilance analyses with interpretable denominators or comparator context, and (iv) phenotype-defining case reports or series, particularly those supported by urinary indices, kidney biopsy, or both (Powles et al., 2021; Bardia et al., 2021; Guarin et al., 2024; Moore et al., 2023; Kalantri and Lomashvili, 2023; Cortes et al., 2022; Nguyen et al., 2023; Powles et al., 2024; U.S. Food and Drug Administration, 2025a; U.S. Food and Drug Administration, 2025b; U.S. Food and Drug Administration, 2025c; U.S. Food and Drug Administration, 2024).

The goal of this review was not to generate a one-dimensional ranking of CKD risk by drug. Instead, we sought to organize heterogeneous kidney and electrolyte safety signals into a framework that links drug design and intracellular handling to syndrome-level renal phenotypes. For that reason, each major ADC subsection begins with the drug’s pharmacologic background (target, linker-payload class, approved indication(s), and dosing), then moves to trial-level evidence, label/regulatory data, and finally phenotype-defining case evidence.

### 2.2 Outcome definitions and operational KDIGO mapping

Kidney outcomes reported in oncology trials are heterogeneous. Many studies use terms such as “renal impairment,” “creatinine increased,” or general laboratory categories rather than KDIGO-defined acute kidney injury (AKI) endpoints. We, therefore, specify the following operational rules for standardization (Khwaja, 2012; Kellum et al., 2013):

1. KDIGO AKI staging was applied only when supportable from the source report. When baseline serum creatinine and the relevant time course were available, renal events were interpreted using KDIGO AKI criteria based on serum creatinine.
2. Urine-output criteria were not applied. These data are rarely available in trial reports, labels, or case literature for ADCs.
3. Non-specific trial terminology was preserved when staging was not supportable. If a study reported only “renal impairment” or “creatinine increased” without sufficient baseline or timing information, we retained the original terminology and did not force a KDIGO stage assignment.
4. AKD or CKD terminology was used only when chronicity was explicit. We did not infer chronic kidney disease from isolated creatinine elevations.
5. Electrolyte abnormalities were extracted as reported. We preserved the source definition (e.g., CTCAE grade or laboratory threshold) while emphasizing clustered patterns that localize injury to nephron segments, such as phosphate wasting with normoglycemic glycosuria and non-anion-gap metabolic acidosis.

This explicit approach was adopted to avoid false precision while still improving cross-study interpretability.

### 2.3 Evidence hierarchy and role of case reports

To minimize inappropriate “class effect” inference, claims are presented using explicit evidence hierarchy labeling by source type:

- Level A: randomized or registrational trial evidence (Powles et al., 2021; Bardia et al., 2021; Moore et al., 2023; Cortes et al., 2022; Powles et al., 2024)
- Level B: label or regulatory summaries (U.S. Food and Drug Administration, 2025a; U.S. Food and Drug Administration, 2025b; U.S. Food and Drug Administration, 2025c; U.S. Food and Drug Administration, 2024)
- **Level C:** real-world cohorts or pharmacovigilance analyses with clinically interpretable context
- **Level D:** case reports or series, given higher weight when urinary indices or biopsies support the mechanism (Guarin et al., 2024; Kalantri and Lomashvili, 2023)
- Level E: mechanistic inference, preclinical extrapolation, or biologic plausibility (Metrangolo and Engelholm, 2024; Nguyen et al., 2023; Zhao et al., 2018; Tarantino et al., 2022; Shim, 2020; Ogitani et al., 2016; Christensen and Birn, 2001; Giugl et al., 2022; Caculitan et al., 2017; Suzuki et al., 2019; Shirahama-Noda et al., 2003; Kenny, 1977; Kaushal et al., 2013; Jain et al., 2015; Erickson et al., 2006; Doronina et al., 2003; Eshbach and Weisz, 2017; Rbaibi et al., 2023; Morita et al., 2007; Madsen and Park, 1987; Ryman and Meibohm, 2017; Ovacik and Lin, 2018)

Case reports are, therefore, used here to refine high-specificity phenotype hypotheses, not to estimate population incidence. When case-level details were available, we incorporated patient characteristics, tumor setting, time-to-onset, urinary findings, biopsy results, and reversibility into the narrative so that the reader can judge the strength and limits of the evidence more directly.

A detailed trial-, label-, and case-level renal/electrolyte evidence extraction matrix is provided in Supplementary Table S1 to make the evidence hierarchy, original renal terminology, electrolyte reporting, tumor context, dosing schedule, comparator/sample information, exposure or follow-up when reported, and KDIGO mappability transparent.

## 3 ADC platform background relevant to renal safety

ADCs are structurally heterogeneous, and a pharmacology-oriented review must, therefore, identify the platform features most relevant to renal exposure before discussing renal phenotypes. These features include target antigen, antibody class, linker chemistry, payload class, drug-to-antibody ratio, membrane permeability of released species, and approved dosing schedule (Metrangolo and Engelholm, 2024; Nguyen et al., 2023; Tarantino et al., 2022; Aggarwal et al., 2023; Maecker et al., 2023). The major solid-tumor ADCs emphasized in this review are summarized in Table 1.

**TABLE 1:** Major solid-tumor ADCs discussed in this review: pharmacologic features, typical dosing, and renal relevance.

ADC	Platform features	Typical solid-tumor use and dose	Renal and electrolyte relevance
Enfortumab vedotin	Nectin-4-directed; cleavable mc-vc-PABC linker; monomethyl auristatin E (MMAE) payload	Advanced urothelial carcinoma; 1.25 mg/kg IV on days 1, 8, and 15 of a 28-day cycle	Systemic hyperglycemia, diabetic ketoacidosis (DKA), infection, and dehydration can drive prerenal AKI; label also notes creatinine, phosphate, and potassium abnormalities
Sacituzumab govitecan	Trop-2-directed; hydrolysable linker; SN-38 payload	Metastatic triple-negative breast cancer (TNBC) and HR-positive/HER2-negative metastatic breast cancer; 10 mg/kg IV on days 1 and 8 of a 21-day cycle	Diarrhea and dehydration dominate the renal signal; rare biopsy-proven acute tubulointerstitial nephritis (ATIN) expands the differential diagnosis
Mirvetuximab soravtansine	FRalpha-directed; cleavable disulfide linker; DM4 payload	FRalpha-positive platinum-resistant ovarian cancer; 6 mg/kg adjusted ideal body weight every 3 weeks	Kidney-specific data remain sparse; improved global tolerability does not exclude mild or electrolyte-predominant injury
Trastuzumab deruxtecan	HER2-directed; cleavable tetrapeptide linker; membrane-permeable DXd payload	5.4 mg/kg every 3 weeks for most solid-tumor settings; 6.4 mg/kg every 3 weeks in gastric cancer	Hypothesis-defining Fanconi-like signal; label highlights limited pharmacokinetic data in severe renal impairment

### 3.1 Enfortumab vedotin

Enfortumab vedotin (EV) is a nectin-4-directed ADC that uses a protease-cleavable maleimidocaproyl-valine-citrulline linker to deliver the microtubule toxin monomethyl auristatin E (MMAE) (Tarantino et al., 2022; Aggarwal et al., 2023; U.S. Food and Drug Administration, 2025c). In urothelial carcinoma, the approved EV dose is 1.25 mg/kg intravenously on days 1, 8, and 15 of a 28-day cycle, either as monotherapy or in combination regimens, depending on disease setting (U.S. Food and Drug Administration, 2025c). From a renal perspective, EV is relevant because its clinical program prominently includes systemic toxicities, especially hyperglycemia and rare diabetic ketoacidosis, that can secondarily precipitate AKI.

### 3.2 Sacituzumab govitecan

Sacituzumab govitecan (SG) is a Trop-2-directed ADC that delivers the topoisomerase I inhibitor SN-38 through a hydrolyzable linker (Tarantino et al., 2022; Aggarwal et al., 2023; U.S. Food and Drug Administration, 2025b). The approved dose is 10 mg/kg intravenously on days 1 and 8 of a 21-day cycle (U.S. Food and Drug Administration, 2025b). In solid tumors, the current label includes metastatic triple-negative breast cancer and hormone receptor-positive/HER2-negative metastatic breast cancer. Pharmacologically, SG is important in this review because diarrhea and dehydration are prominent on-target/off-tumor toxicities at the whole-patient level, while a rare biopsy-proven immune-interstitial phenotype has also been described (Guarin et al., 2024; U.S. Food and Drug Administration, 2025b).

### 3.3 Mirvetuximab soravtansine

Mirvetuximab soravtansine is a folate receptor-alpha (FRalpha)-directed ADC carrying the maytansinoid payload DM4 through a cleavable disulfide linker (Moore et al., 2023; Tarantino et al., 2022; Aggarwal et al., 2023; U.S. Food and Drug Administration, 2024). In FRalpha-positive, platinum-resistant ovarian cancer, the FDA-approved dose is 6 mg/kg based on adjusted ideal body weight every 3 weeks (U.S. Food and Drug Administration, 2024). Kidney-specific comparative data are limited, but the agent is useful conceptually because its global tolerability advantage over chemotherapy does not eliminate the need for kidney and electrolyte vigilance.

### 3.4 Trastuzumab deruxtecan

Trastuzumab deruxtecan (T-DXd) is a HER2-directed ADC with an enzymatically cleavable tetrapeptide linker and a membrane-permeable topoisomerase I inhibitor payload (DXd) (Metrangolo and Engelholm, 2024; Tarantino et al., 2022; Aggarwal et al., 2023; U.S. Food and Drug Administration, 2025a). The approved solid-tumor dose is 5.4 mg/kg every 3 weeks for most breast and other solid-tumor indications and 6.4 mg/kg every 3 weeks for HER2-positive gastric cancer (U.S. Food and Drug Administration, 2025a). T-DXd is particularly relevant to the discussion of renal phenotype because it combines a payload capable of bystander diffusion with a published Fanconi-like case signal and limited pharmacokinetic data in severe renal impairment (Kalantri and Lomashvili, 2023; U.S. Food and Drug Administration, 2025a).

## 4 Mechanistic basis of renal handling and injury

### 4.1 Biophysical basis of renal exposure: filtration constraint versus tubular uptake

The starting point for kidney interpretation is molecular size. ADCs are built on an IgG backbone of roughly 150 kDa, and intact molecules are, therefore, expected to undergo minimal glomerular filtration when the filtration barrier is preserved (Metrangolo and Engelholm, 2024; Nguyen et al., 2023; Ryman and Meibohm, 2017; Ovacik and Lin, 2018). This biophysical constraint makes the high-flux luminal entry of intact ADCs unlikely. In contrast, many payloads or payload-containing catabolites are small molecules, often below 1 kDa, and become more readily filterable when present in the systemic circulation (Metrangolo and Engelholm, 2024; Tarantino et al., 2022; Jain et al., 2015; Erickson et al., 2006). As a result, intact ADCs and circulating payload species should be treated as pharmacologically distinct renal exposures rather than as a single entity.

**FIGURE 1:**
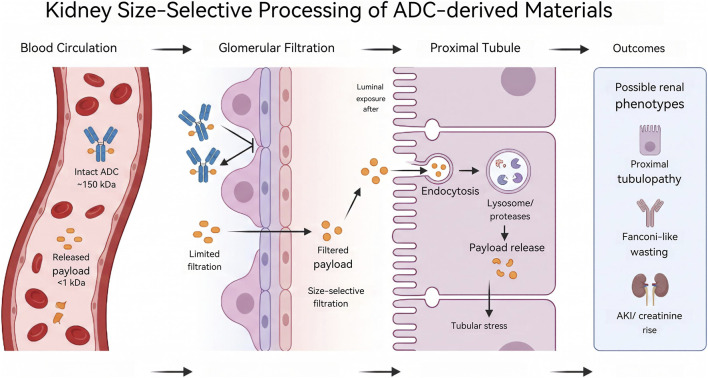
Size-selective renal handling and proximal tubular processing of ADC-derived materials.

For intact ADCs that reach the kidney from the peritubular circulation, plausible exposure routes include fluid-phase endocytosis, noncanonical receptor-mediated uptake, endothelial handling, or macropinocytosis. This interpretation draws on ADC off-target uptake literature, antibody pharmacokinetics, and proximal tubule/endothelial cell biology rather than direct kidney-specific ADC tracing studies (Zhao et al., 2018; Christensen and Birn, 2001; Eshbach and Weisz, 2017; Rbaibi et al., 2023; Ryman and Meibohm, 2017; Ovacik and Lin, 2018). For released small-molecule payloads or membrane-permeable catabolites, the dominant route can instead become glomerular filtration followed by luminal exposure and proximal tubular entry, potentially mediated by apical transporters or protein-reclamation systems such as megalin/cubilin for selected species (Metrangolo and Engelholm, 2024; Tarantino et al., 2022; Christensen and Birn, 2001; Eshbach and Weisz, 2017; Rbaibi et al., 2023). This size-selective filtration gate is central to the framework proposed here and is summarized conceptually in Figure 1.

This schematic illustrates a conceptual framework for the renal handling of ADC-derived material. Intact ADCs are large IgG-based macromolecules (approximately 150 kDa) and are, therefore, expected to undergo minimal glomerular filtration under an intact filtration barrier. In contrast, released payloads or payload-containing catabolites are substantially smaller and may become filterable, thereby creating luminal exposure within the proximal tubule. Following filtration, these small ADC-derived species may enter, accumulate in, or be processed by proximal tubular cells, where endolysosomal proteases and related intracellular processing pathways may contribute to tubular stress. The downstream clinical consequences may include proximal tubulopathy, Fanconi-like wasting, and AKI manifested by an increase in serum creatinine. This figure is intended as a mechanistic and conceptual model; direct kidney-specific evidence for several of these pathways remains limited.

### 4.2 Renal proteolytic environment: ADC intracellular processing is not a single pathway

ADC processing is often described too simplistically as though a single lysosomal protease in a single compartment determines payload release. That formulation is misleading. Foundational ADC studies show that antibody–maytansinoid conjugates require lysosomal degradation and linker-dependent intracellular processing to generate active metabolites, while auristatin ADCs were designed around intracellular protease-cleavable linkers (Erickson et al., 2006; Doronina et al., 2003). More recent work showed that protease-cleavable linkers such as valine-citrulline can be processed by overlapping protease networks and that cathepsin B can be dispensable for the cellular processing of cathepsin B-cleavable ADCs in several experimental systems (Caculitan et al., 2017; Jain et al., 2015). Put differently, linker cleavage should be modeled as a protease-redundant intracellular network rather than as a single enzyme controlling one obligatory step.

This point may be particularly relevant in the kidney because the proximal tubule is a highly endocytic epithelial system specialized for the reclamation and degradation of filtered proteins and small bioactive molecules (Christensen and Birn, 2001; Eshbach and Weisz, 2017; Rbaibi et al., 2023). Classic renal work mapped cathepsin B and L activity across microdissected proximal tubular segments and showed that the lysosomal-vacuolar system is most prominent in the S1 segment, a major site of protein reabsorption (Madsen and Park, 1987). Later studies showed that cathepsin D is enriched in proximal tubular epithelial cells and that asparaginyl endopeptidase (AEP, legumain) is highly expressed in late endosomes and lysosomes of renal proximal tubular cells and participates in lysosomal protease processing (Suzuki et al., 2019; Shirahama-Noda et al., 2003; Morita et al., 2007). These observations do not prove an ADC nephrotoxicity mechanism, but they strongly support the biologic plausibility that renal tubular cells possess the intracellular machinery required to process internalized ADC-derived material.

In addition to intracellular lysosomal proteases, the proximal tubular apical brush border is itself a proteolytically active interface. Classical biochemical studies identified endopeptidase activities in the renal proximal tubule microvillus membrane, including neutral endopeptidase and dipeptidyl peptidase activity, while meprin-family metalloproteinases are abundant at the apical membranes of proximal tubules and have been implicated in ischemia-reperfusion and cisplatin-associated AKI biology (Kenny, 1977; Kaushal et al., 2013). These observations strengthen the concept that the proximal tubule is not a passive recipient of filtered ADC-derived material but a surface- and endolysosome-rich epithelial processing compartment. However, brush-border enzymes should not be interpreted as a demonstrated route of intact ADC entry, and whether these enzymes cleave clinically relevant ADC linkers or payload-containing catabolites *in vivo* remains unproven.

Kidney-specific studies have not yet directly demonstrated local intrarenal proteolytic processing as the dominant driver of ADC-associated nephrotoxicity. The available evidence is stronger for a chain of inference: cleavable linkers, intracellular catabolic redundancy, lysosomal degradation, and systemic free-payload exposure matter in ADC toxicology broadly, and renal tubular cells are biologically equipped for high-capacity endocytosis and endolysosomal proteolysis (Caculitan et al., 2017; Jain et al., 2015; Erickson et al., 2006; Doronina et al., 2003; Eshbach and Weisz, 2017; Rbaibi et al., 2023; Morita et al., 2007; Madsen and Park, 1987; Ryman and Meibohm, 2017; Ovacik and Lin, 2018). We, therefore, frame renal proteolytic processing as a biologically plausible amplifier of kidney exposure rather than a proven class mechanism. This distinction is important to prevent over-interpretation of limited case-based signals.

### 4.3 Uptake routes beyond target-mediated endocytosis

ADC toxicology cannot be understood by target expression alone. Non-specific uptake routes, including fluid-phase endocytosis and macropinocytosis, can contribute to off-target tissue exposure in settings with high endocytic activity (Zhao et al., 2018). Direct evidence for these routes in human renal parenchyma remains limited, but the proximal tubular and endothelial compartments are biologically compatible with such uptake because proximal tubular cells elaborate a high-capacity apical endocytic pathway for filtered ligands (Christensen and Birn, 2001; Eshbach and Weisz, 2017; Rbaibi et al., 2023). This is particularly relevant when renal toxicity does not map cleanly to target antigen distribution.

### 4.4 Linker stability and systemic shedding as renal determinants

Linker chemistry functions as a form of safety-by-design. Greater systemic stability should reduce the circulating pool of free-payload or small-payload-containing catabolites that would otherwise acquire a filtration advantage (Metrangolo and Engelholm, 2024; Nguyen et al., 2023; Tarantino et al., 2022; Jain et al., 2015). Conversely, premature extracellular cleavage, hydrolysis, or formation of membrane-permeable catabolites can increase renal luminal exposure even if the intact ADC itself scarcely filters. Lysosomal degradation and linker-dependent intracellular processing also determine which active or residual payload species reach intracellular targets after uptake (Erickson et al., 2006; Doronina et al., 2003). This is why renal safety should not be inferred from antibody specificity alone; linker behavior and payload physicochemical properties can be equally important.

### 4.5 Lysosomal escape transporters and intracellular exposure

Transporters such as SLC46A3 influence the egress of linker-payload catabolites from lysosomes, particularly for non-cleavable maytansinoid ADCs (Hamblett et al., 2015). Together with lysosomal degradation and linker-dependent catabolism, lysosomal escape can theoretically modulate both efficacy and tissue-specific toxicity (Hamblett et al., 2015; Erickson et al., 2006). For the kidney, this remains hypothesis-generating, but it provides a plausible mechanistic layer for inter-tissue and inter-patient heterogeneity that cannot be explained solely by antigen expression or plasma exposure.

## 5 Comparative clinical evidence: trial-first synthesis

### 5.1 Enfortumab vedotin: a predominantly systemic-to-renal AKI axis

EV has direct comparative evidence against chemotherapy in advanced urothelial carcinoma. In EV-301, patients with previously treated advanced urothelial carcinoma received EV 1.25 mg/kg on days 1, 8, and 15 of a 28-day cycle and were compared with investigator-chosen chemotherapy (Powles et al., 2021). Longer follow-up and subsequent frontline studies, such as EV-302, further reinforce the broader point that ADCs redistribute toxicity rather than uniformly eliminate severe adverse events or treatment discontinuations (Powles et al., 2021; Powles et al., 2024).

For renal interpretation, EV is notable not because it has the clearest direct tubular signature, but because it illustrates how systemic toxicities can dominate kidney outcomes. Hyperglycemia, diabetic ketoacidosis, infection, and poor oral intake can produce osmotic diuresis, dehydration, and prerenal AKI (Powles et al., 2021; U.S. Food and Drug Administration, 2025c). Consistently, the current U.S. prescribing information lists AKI among serious adverse reactions in key regimens and also reports laboratory abnormalities, including increased creatinine and decreased phosphate or potassium (U.S. Food and Drug Administration, 2025c). In practice, EV-associated creatinine rise should therefore not be assumed to represent a uniform direct tubular program. The more likely differential often begins with systemic toxicity, hemodynamics, and concurrent nephrotoxins.

### 5.2 Sacituzumab govitecan: gastrointestinal volume depletion plus a rare interstitial phenotype

In the ASCENT trial, adults with relapsed or refractory metastatic triple-negative breast cancer received SG 10 mg/kg on days 1 and 8 of 21-day cycles *versus* physician’s-choice chemotherapy (Bardia et al., 2021). The current label also includes hormone receptor-positive/HER2-negative metastatic breast cancer, but the ASCENT trial remains the most direct randomized chemotherapy comparison anchored in our review (Bardia et al., 2021; U.S. Food and Drug Administration, 2025b). Across studies and regulatory summaries, the dominant toxicity axis is gastrointestinal, especially diarrhea, which can lead to dehydration, electrolyte loss, and prerenal AKI (Bardia et al., 2021; U.S. Food and Drug Administration, 2025b).

SG also has one of the clearest high-specificity kidney case signals in the ADC literature. A 51-year-old woman with metastatic ER-positive/PR-positive/HER2-negative breast cancer developed vomiting, diarrhea, nephrotic-range proteinuria, severe AKI requiring hemodialysis, and biopsy-proven ATIN after SG exposure; kidney function improved with corticosteroids, supporting an immune-interstitial phenotype rather than a purely prerenal event (Guarin et al., 2024). This case does not establish incidence, but it materially changes the differential diagnosis: in an SG-treated patient with persistent AKI, disproportionate to the apparent volume deficit, or accompanied by sterile pyuria/inflammatory features, ATIN should be actively considered.

### 5.3 Mirvetuximab soravtansine: improved global tolerability does not equal absence of kidney risk

Mirvetuximab soravtansine was compared with chemotherapy in patients with FRalpha-positive, platinum-resistant ovarian cancer in the MIRASOL trial, using 6 mg/kg adjusted ideal body weight every 3 weeks (Moore et al., 2023; U.S. Food and Drug Administration, 2024). Compared with conventional chemotherapy, the drug shows a more favorable global tolerability profile for several classic cytotoxic toxicities (Moore et al., 2023). However, the renal lesson is one of interpretive caution: reduced rates of overt traditional toxicity do not prove the absence of renal injury, especially when renal and electrolyte end points are not deeply phenotyped.

In other words, a negative creatinine signal in a trial should not automatically be read as proof of renal neutrality. For ADCs, renal injury may be mild, segment-selective, electrolyte-predominant, or partially masked by preserved renal functional reserve. Accordingly, mirvetuximab is useful in this framework as an example of why pharmacologic plausibility and monitoring intensity still matter even when global tolerability appears to be improved.

### 5.4 Trastuzumab deruxtecan: a hypothesis-defining proximal tubule signal

T-DXd has direct comparative evidence against chemotherapy-based ADC comparators, most notably in DESTINY-Breast03, where patients with HER2-positive metastatic breast cancer received T-DXd 5.4 mg/kg every 3 weeks *versus* trastuzumab emtansine (Cortes et al., 2022). Yet the most striking renal signal is not derived from trial incidence tables but from a phenotype-defining case report. A 74-year-old woman with metastatic recurrent ER-positive/PR-negative/HER2-positive breast cancer developed hypophosphatemia, hypokalemia, hypomagnesemia, non-anion-gap metabolic acidosis, glycosuria with normoglycemia, and urinary phosphate and potassium wasting while on trastuzumab deruxtecan; the syndrome reversed after withdrawal, supporting drug-associated Fanconi syndrome (Kalantri and Lomashvili, 2023).

This pattern is highly informative because it localizes injury to the proximal tubule and may precede an overt increase in creatinine. It is, therefore, better interpreted as a nephron-segment signal than as a generic “renal impairment” event. The current label also highlights an important evidence gap: pharmacokinetics in severe renal impairment remain insufficiently characterized, and most available data come from patients with mild-to-moderate impairment (U.S. Food and Drug Administration, 2025a). As such, T-DXd currently represents the clearest hypothesis-generating ADC signal for proximal tubulopathy but not yet a defined population incidence program.

## 6 Benchmarking against conventional chemotherapy: canonical renal-electrolyte programs

### 6.1 Platinum agents

Platinum agents remain the reference standard for mechanism-coherent chemotherapy nephrotoxicity. Cisplatin enters tubular cells through renal transport pathways and triggers oxidative stress, inflammation, and tubular injury (Romani, 2015; Saito et al., 2017; Bhat et al., 2015). Importantly, cisplatin-associated AKI has a comparatively mature prevention literature, including systematic evaluation of preventive strategies, reinforcing its value as a clinically monitorable benchmark for mechanism-directed nephrotoxicity prevention (Hamroun et al., 2019). Clinically, renal magnesium wasting is highly reproducible and may persist long after exposure, often accompanied by secondary hypokalemia and occasionally hypocalcemia (Romani, 2015; Nardelli et al., 2025; Ledeganck et al., 2013). Downregulation of the EGF–TRPM6 axis in the distal convoluted tubule provides a segment-specific explanation for this electrolyte signature (Ledeganck et al., 2013).

### 6.2 High-dose methotrexate

HDMTX exemplifies a different program: intratubular precipitation and crystal nephropathy with abrupt AKI and delayed methotrexate clearance (Howard et al., 2016; Widemann et al., 2014; Ramsey et al., 2018). This phenotype is distinct because it is strongly amenable to mechanism-directed prevention and rescue, including hydration, urine alkalinization, leucovorin escalation, and glucarpidase when indicated (Howard et al., 2016; Widemann et al., 2014; Ramsey et al., 2018). It, therefore, serves as a useful benchmark for phenotype-aligned management.

### 6.3 Ifosfamide

Ifosfamide remains the classic comparator for proximal tubular dysfunction and acquired Fanconi syndrome. Experimental data support chloroacetaldehyde as a major mediator of proximal tubular mitochondrial toxicity and generalized solute wasting (Zamlauski-Tucker et al., 1994). When interpreting rare ADC-associated Fanconi-like patterns, ifosfamide provides the clearest benchmark for urinary phosphate wasting, normoglycemic glycosuria, bicarbonate wasting, and preserved or only modestly impaired GFR early in the course.

### 6.4 Gemcitabine and anti-VEGF therapy

Microvascular and endothelial kidney injury is best benchmarked against established programs such as gemcitabine-associated thrombotic microangiopathy (TMA) and anti-VEGF-associated proteinuric glomerular injury (Uyl et al., 2025; Izzedine and Perazella, 2015; Izzedine et al., 2006; Person et al., 2019). These syndromes are uncommon but high-stakes, and they remind the clinician that a proteinuric, hypertensive, hemolytic picture should not be casually collapsed into generic AKI. This benchmark is especially important when evaluating possible ADC-associated TMA-like signals that lack syndrome-defining laboratory or histopathologic support.

### 6.5 Combination regimens and attribution challenges

Combination therapy complicates causal attribution. Pemetrexed, immunotherapy combinations, platinum backbones, and prior nephrotoxic exposure can all blur the mechanistic clarity that is more obvious in textbook single-agent toxicology (Glezerman et al., 2011; Gilad et al., 2021). This same problem applies to ADC practice, particularly as newer ADC regimens move earlier in treatment lines and are paired with immune checkpoint inhibitors or prior platinum exposure.

## 7 Pattern-informed renal phenotypes: a trafficking-aligned taxonomy

The practical value of a mechanism-informed review lies in whether it sharpens syndrome recognition. We, therefore, propose a trafficking-aligned taxonomy that connects ADC processing and exposure hypotheses to renal phenotype clusters defined by syndrome, electrolyte pattern, and diagnostic discriminators. The goal is not to assign certainty where evidence is limited but to make differential diagnosis more explicit and more biologically grounded. Table 2 summarizes the mechanistic and phenotypic benchmarking of ADC-associated renal signals against canonical chemotherapy programs.

**TABLE 2:** Mechanistic and phenotypic benchmarking of ADC-associated renal signals against canonical chemotherapy programs.

Agent or program	Major renal phenotype	Signature clues	Mechanistic interpretation	Evidence
Cisplatin	Tubular injury with magnesium wasting	Persistent hypomagnesemia; urinary Mg wasting; secondary hypokalemia ± hypocalcemia	Transporter-mediated tubular uptake and distal EGF–TRPM6 downregulation	A/B
HDMTX	Crystal nephropathy	Abrupt AKI, delayed methotrexate clearance, crystalluria	Intratubular precipitation with secondary tubular injury	A/B
Ifosfamide	Proximal tubulopathy/Fanconi syndrome	Low phosphate and potassium, glycosuria with normal serum glucose, bicarbonate wasting	Chloroacetaldehyde-driven proximal tubular mitochondrial toxicity	A/B
Gemcitabine/anti-VEGF	TMA or proteinuric endothelial injury	Hypertension, proteinuria, thrombocytopenia, hemolysis	Microvascular and endothelial injury program	A/B
Enfortumab vedotin	Predominantly prerenal/systemic AKI	Hyperglycemia, ketones or DKA, dehydration, rapid improvement after fluids	Systemic metabolic crisis and volume depletion dominate the renal presentation	A/B
Sacituzumab govitecan	Prerenal AKI ± ATIN	Diarrhea-linked AKI; if persistent, sterile pyuria, proteinuria, or biopsy ATIN	GI toxicity and dehydration, with rare immune-interstitial injury	A/B/D
Mirvetuximab soravtansine	Under-characterized renal signal	Limited creatinine signal; renal endpoints under-ascertained	Distinct adverse event (AE) spectrum, but the kidney phenotype may be mild or missed	A/B/E
Trastuzumab deruxtecan	Proximal tubulopathy/Fanconi-like syndrome	Hypophosphatemia, normoglycemic glycosuria, non-anion-gap acidosis, low Mg/K	Hypothesized luminal exposure to filterable payload species with proximal tubular vulnerability	D/E

### 7.1 Phenotype cluster 1: proximal tubulopathy/Fanconi-like syndrome

The hallmark pattern is phosphate wasting with normoglycemic glycosuria, non-anion-gap metabolic acidosis, and, frequently, hypokalemia and hypomagnesemia. Low-molecular-weight proteinuria or elevated fractional excretion of phosphate/magnesium can strengthen localization to the proximal tubule (Kalantri and Lomashvili, 2023). This phenotype currently has its clearest ADC anchor in the T-DXd case literature and its classical chemotherapy anchor in ifosfamide (Kalantri and Lomashvili, 2023; Zamlauski-Tucker et al., 1994).

### 7.2 Phenotype cluster 2: prerenal AKI driven by systemic toxicity

This phenotype is dominated by volume depletion, infection, sepsis, or metabolic crises such as diabetic ketoacidosis. It is often accompanied by electrolyte abnormalities, but the renal dysfunction is secondary to systemic illness rather than primary tubular injury (Powles et al., 2021; Bardia et al., 2021; Khwaja, 2012; U.S. Food and Drug Administration, 2025c). EV and SG are particularly important here because their prominent systemic toxicities can produce clinically meaningful AKI without requiring direct renal cytotoxicity as the principal mechanism.

### 7.3 Phenotype cluster 3: acute tubulointerstitial nephritis

ATIN should be suspected when AKI persists despite supportive care, accompanied by sterile pyuria, inflammatory features, mild proteinuria, or biopsy evidence (Guarin et al., 2024). At present, SG provides the most concrete example of ADC-associated disease through a biopsy-proven case report (Guarin et al., 2024). The key point is diagnostic humility: diarrhea-associated AKI and ATIN can coexist or sequentially confound one another.

### 7.4 Phenotype cluster 4: microvascular/endothelial program

This cluster includes AKI with hemolysis, thrombocytopenia, hypertension, significant proteinuria, or both. Robust benchmarks exist in the chemotherapy and anti-VEGF literature, but ADC-specific causality remains far less mature (Uyl et al., 2025; Izzedine and Perazella, 2015; Izzedine et al., 2006; Person et al., 2019). In this framework, TMA is best treated as a rule-in/rule-out high-stakes differential diagnosis rather than a frequently established ADC class effect.

## 8 Translational implications for pharmacovigilance, monitoring, and endpoint design

### 8.1 Baseline vulnerability and “second-hit” biology

Many patients who receive modern ADCs do not enter treatment with a pristine kidney. Prior platinum exposure, prior AKI, cumulative tubular injury, diabetes, hypertension, obesity, diuretics, SGLT2 inhibitors, renin–angiotensin system blockers, and baseline occult glycosuria or proteinuria all alter the kidney’s reserve and may amplify later toxicity signals. This is especially important for electrolyte-predominant phenotypes, in which an ADC may unmask or amplify pre-existing tubular vulnerability rather than create a *de novo* syndrome. Table 3 summarizes baseline vulnerability domains and practical actions during ADC therapy.

**TABLE 3:** Baseline stratification and second-hit vulnerability domains during ADC therapy.

Vulnerability domain	Baseline assessment	Why it matters during ADC therapy	Practical action
Reduced proximal tubular reserve	Urinalysis for glycosuria or low-molecular-weight proteinuria; baseline phosphate and bicarbonate; prior ifosfamide or cisplatin	May unmask earlier or more severe Fanconi-like phenotypes during payload exposure to the tubular lumen	Intensify ion-first surveillance and correct baseline electrolyte abnormalities before infusion
Distal electrolyte vulnerability	Baseline magnesium and urinary magnesium wasting; prior platinum exposure	Diarrhea-prone ADCs may convert latent magnesium deficit into refractory hypomagnesemia and secondary hypokalemia	Use proactive magnesium replacement and a lower threshold for intravenous rehydration
Endothelial/microvascular vulnerability	Blood pressure, urine protein-creatinine ratio, platelet count, and hemolysis screen; prior gemcitabine or anti-VEGF therapy	Lowers the threshold for considering TMA when proteinuria, hypertension, or hemolysis emerges	Close blood pressure and proteinuria surveillance; urgent TMA work-up when indicated
Hemodynamic fragility	Diuretics, SGLT2 inhibitors, renin-angiotensin system blockers, baseline hydration status	Amplifies prerenal AKI during EV-associated hyperglycemia/DKA or SG-associated diarrhea	Dynamic medication adjustment and aggressive sick-day counseling around treatment

### 8.2 On-treatment monitoring: ion-first surveillance and targeted tubular work-up

For ADC programs with plausible proximal tubular involvement or prominent systemic volume-depleting toxicity, an “ion-first” strategy remains more informative than relying on creatinine alone. During at least the first three treatment cycles of higher-risk programs, we suggest routine monitoring of serum magnesium, phosphate, potassium, bicarbonate, and urinalysis (with attention to glucose and protein) in addition to serum creatinine/eGFR (KDIGO, 2020; Romani, 2015; Kalantri and Lomashvili, 2023; Nardelli et al., 2025; Khwaja, 2012).

When an electrolyte cluster emerges, further targeted evaluation becomes more valuable than repeating creatinine alone. Fractional excretion of phosphate and magnesium, urine glucose with simultaneous serum glucose, and low-molecular-weight urinary proteins such as beta2-microglobulin can help distinguish proximal tubulopathy from prerenal confounding (Kalantri and Lomashvili, 2023). Conversely, when AKI does not improve with volume repletion or is accompanied by inflammatory urine sediment, the work-up should pivot toward ATIN.

### 8.3 Subclinical and tubular injury endpoints

Standard clinical measures of kidney function, including serum creatinine and cystatin C, primarily capture filtration performance rather than structural nephron injury. Because renal functional reserve can buffer mild or focal tubular damage, stable creatinine or cystatin C values do not prove the absence of kidney injury (Haase et al., 2011; Strader et al., 2025). The broader AKI literature established the concept of biomarker-positive, “subclinical AKI,” in which damage biomarkers become abnormal before diagnostic loss of filtration function is captured by creatinine-based criteria (Haase et al., 2011).

For ADC programs, this distinction is especially relevant because injury may be electrolyte-predominant, proximal tubule-selective, or mild enough to remain functionally silent early on. Urinary kidney injury molecule-1 (KIM-1) and urinary neutrophil gelatinase-associated lipocalin (NGAL) are not yet validated ADC-specific trial end points, but they are biologically attractive complementary markers of tubular injury and deserve prospective study in high-risk populations such as patients with diabetes, hypertension, obesity, prior platinum or ifosfamide exposure, or baseline evidence of reduced tubular reserve (Haase et al., 2011; Strader et al., 2025). Experience from cisplatin programs further supports the principle that urinary damage biomarkers can detect subclinical nephrotoxicity when serum creatinine remains unchanged (Strader et al., 2025). Accordingly, we view urinary KIM-1 and NGAL as exploratory renal safety end points for future ADC trials rather than as current standards of care.

### 8.4 Event-driven diagnostic algorithm

**FIGURE 2:**
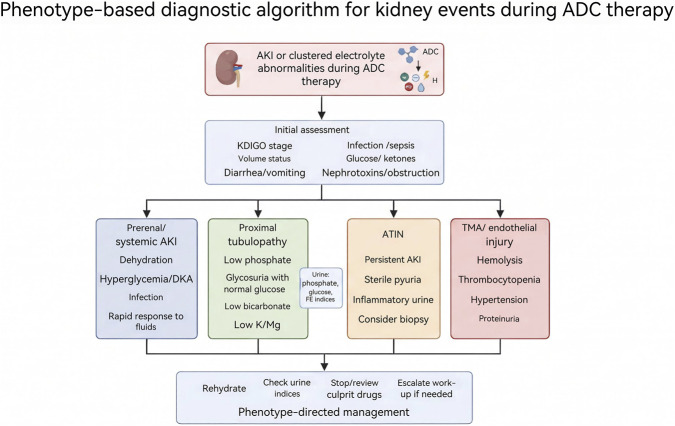
Phenotype-based diagnostic algorithm for kidney events during ADC therapy. This schematic outlines a phenotype-based clinical approach to acute kidney injury (AKI) or clustered electrolyte abnormalities arising during ADC therapy. Initial assessment includes AKI severity staging, volume status, gastrointestinal losses, infection or sepsis, glucose and ketone assessment, nephrotoxin exposure, and urinary obstruction. The subsequent evaluation is organized into four major clinical phenotypes: (1) prerenal or systemic AKI, often associated with dehydration, hyperglycemia/diabetic ketoacidosis (DKA), or infection; (2) proximal tubulopathy, suggested by hypophosphatemia, glycosuria with normal blood glucose, low bicarbonate, and associated potassium or magnesium wasting; (3) acute tubulointerstitial nephritis (ATIN), suggested by persistent AKI, sterile pyuria, and inflammatory urinary sediment; and (4) thrombotic microangiopathy (TMA) or endothelial injury, suggested by hemolysis, thrombocytopenia, hypertension, and proteinuria. The figure emphasizes phenotype-directed management, including rehydration, urine-based tubular indices when appropriate, review and holding of suspected drugs, and escalation of diagnostic evaluation when clinically indicated.

When AKI or clustered electrolyte abnormalities emerge during ADC therapy, a phenotype-based algorithm can sharpen differential diagnosis. Initial steps should standardize severity with KDIGO terminology when possible and then assess hemodynamics, volume status, diarrhea, infection, hyperglycemia/ketones, concomitant nephrotoxins, and obstruction. If the dominant signal is a clustered electrolyte wasting pattern, work-up should prioritize proximal tubular dysfunction; if hemolysis and thrombocytopenia are present, TMA requires urgent evaluation; and if inflammatory urine findings or failure to respond to supportive care are present, ATIN should move higher on the differential. This logic is summarized in Figure 2.

### 8.5 Proposed minimum core kidney endpoint set for future ADC trials

To move from heterogeneous safety signals to more actionable incidence data, future ADC trials should go beyond non-specific reporting of “renal impairment.” At minimum, higher-risk systemic ADC programs should prospectively report: (i) baseline eGFR, prior cumulative nephrotoxic exposure, baseline qualitative proteinuria, and baseline glycosuria; (ii) serial serum magnesium, phosphate, potassium, bicarbonate, and creatinine during early treatment cycles; (iii) triggered urine biochemistry when clustered electrolyte wasting or grade 2 or higher AKI occurs; and (iv) exploratory tubular injury biomarkers such as urinary KIM-1 and NGAL when feasible in early-phase studies or dedicated renal safety substudies. Such an approach would materially strengthen pharmacovigilance and permit a more mechanistic comparison across linker-payload classes.

## 9 Discussion

The central message of this review is that renal safety in ADC therapy should not be framed as a single drug-ranking exercise. ADC-associated kidney events are better understood as phenotype substitutions or toxicity redistribution shaped by drug design, renal handling, and whole-patient systemic toxicity (Metrangolo and Engelholm, 2024; Nguyen et al., 2023; Tarantino et al., 2022). Intact ADCs and free-payload species do not share the same renal disposition; protease-cleavable linker biology is redundant rather than monolithic; and the kidney itself, especially the proximal tubule, is biologically equipped for high-capacity endocytosis and endolysosomal protein processing (Christensen and Birn, 2001; Caculitan et al., 2017; Suzuki et al., 2019; Shirahama-Noda et al., 2003; Kenny, 1977; Kaushal et al., 2013; Jain et al., 2015; Erickson et al., 2006; Doronina et al., 2003; Eshbach and Weisz, 2017; Rbaibi et al., 2023; Morita et al., 2007; Madsen and Park, 1987).

This perspective also clarifies the role of case reports. The current ADC kidney literature is limited, and trials often under-ascertain renal phenotypes. Yet a biopsy-proven ATIN case or a well-documented Fanconi-like syndrome can still be highly informative when used appropriately. In this review, such cases are treated as phenotype-defining evidence that refines the differential diagnosis and suggests monitoring strategies, not as proof of class-wide incidence.

Several limitations remain. First, renal outcomes are frequently secondary or inconsistently graded in oncology trials (KDIGO, 2020). Second, electrolyte end points remain under-reported. Third, combination regimens, variations in supportive care, and prior nephrotoxic exposure complicate causal attribution. Fourth, severe CKD and dialysis populations remain under-represented in ADC development programs (U.S. Food and Drug Administration, 2025a; U.S. Food and Drug Administration, 2025b; U.S. Food and Drug Administration, 2025c; U.S. Food and Drug Administration, 2024). Finally, although renal proteolytic processing is biologically plausible, direct kidney-specific mechanistic proof remains limited. These gaps are precisely why standardized phenotype ascertainment and biomarker-enriched renal substudies are needed.

## 10 Conclusion

A mechanism-informed, trafficking-aligned framework can reconcile apparently heterogeneous renal safety signals of ADCs by mapping platform design and intracellular processing to renal vulnerability programs and testable phenotype hypotheses. Conventional chemotherapy remains the benchmark for mechanism-coherent renal and electrolyte syndromes, whereas ADCs require closer attention to size-selective filtration constraints, linker stability, non-target uptake, protease-redundant intracellular processing, and payload physicochemical behavior. Clinically, phenotype-first interpretation, especially recognition of proximal tubular electrolyte clusters, immune-interstitial injury, and systemic/prerenal confounding, can improve earlier diagnosis and more rational supportive care. For the next generation of ADC trials, KDIGO-aligned kidney end points, standardized electrolyte reporting, and prospective validation of exploratory tubular injury biomarkers should be treated as priorities rather than afterthoughts.
