# Supplementary material for: From intracellular processing to renal phenotypes: a mechanism-informed framework for interpreting kidney injury and electrolyte patterns with antibody–drug conjugates in solid tumors
Source: Front Pharmacol. 2026 Jun 26;17:1824554. doi: 10.3389/fphar.2026.1824554 (PMC13351378; doi:10.3389/fphar.2026.1824554)
Supplement: Supplementary file 1 [file Table1.docx]

**Supplementary Table S1. Trial-, Label-, and Case-Level Renal/Electrolyte Evidence Matrix**

**Purpose:** This matrix provides drug-, source-, and study-level granularity and avoids reducing heterogeneous renal signals to a single drug-level risk label. Original source terminology is retained where KDIGO staging was not supportable from the source report.

| **Drug / source** | **Evidence type** | **Tumor setting** | **Dose / schedule** | **Comparator / sample** | **Exposure or follow-up** | **Renal endpoint terminology** | **Electrolyte / tubular data** | **KDIGO mappability** | **Framework interpretation** |
| --- | --- | --- | --- | --- | --- | --- | --- | --- | --- |
| Enfortumab vedotin / EV-301 [5] | Level A randomized phase 3 trial | Previously treated locally advanced or metastatic urothelial carcinoma after platinum and PD-1/PD-L1 therapy | EV 1.25 mg/kg IV on days 1, 8, and 15 of a 28-day cycle | Investigator-chosen docetaxel, paclitaxel, or vinflunine; N=608 randomized (301 EV; 307 chemotherapy) | Median follow-up 11.1 months; median treatment duration 5.0 months with EV versus 3.5 months with chemotherapy | Renal events were not deeply phenotyped; interpretation relies on reported adverse-event and laboratory categories | Electrolyte details were not systematically reported as nephron-segment endpoints in the pivotal publication | Generally not KDIGO-mappable unless source-level creatinine timing and baseline values are available | Supports toxicity redistribution and the need to distinguish systemic/prerenal AKI from direct tubular injury |
| Enfortumab vedotin / PADCEV label [40] | Level B regulatory label | Locally advanced or metastatic urothelial carcinoma; monotherapy or combination setting depending on indication | Label-based dosing includes 1.25 mg/kg IV on days 1, 8, and 15 of a 28-day cycle | Regulatory safety summaries from labeled clinical programs | Exposure varies by labeled regimen and line of therapy | AKI listed among serious adverse reactions in key regimens; creatinine increase reported as laboratory abnormality | Laboratory abnormalities include decreased phosphate and potassium in relevant label summaries | Label terms usually not KDIGO-stageable without patient-level creatinine timing | Most consistent with a systemic-to-renal axis involving hyperglycemia/DKA, infection, dehydration, and hemodynamic stress |
| Sacituzumab govitecan / ASCENT [6] | Level A randomized phase 3 trial | Relapsed or refractory metastatic triple-negative breast cancer | SG 10 mg/kg IV on days 1 and 8 of a 21-day cycle | Physician’s-choice chemotherapy; N=529 randomized; safety population 258 SG and 224 chemotherapy | Treatment continued until progression or unacceptable toxicity; heavily pretreated population | Kidney events were not reported as a deeply phenotyped renal endpoint set | Gastrointestinal toxicity is prominent and can secondarily drive volume and electrolyte loss | Not generally KDIGO-mappable from trial tables alone | Anchors the gastrointestinal volume-depletion/prerenal AKI pathway for SG |
| Sacituzumab govitecan / TRODELVY label [39] | Level B regulatory label | Metastatic TNBC and HR-positive/HER2-negative metastatic breast cancer | 10 mg/kg IV on days 1 and 8 of a 21-day cycle | Regulatory safety summaries across labeled programs | Exposure varies by indication and treatment line | Decreased creatinine clearance and diarrhea-related dehydration/AKI are described in label context | Decreased magnesium and potassium may occur, often interpreted with diarrhea and volume depletion | Usually not KDIGO-stageable without patient-level baseline and time course | Supports early fluid and electrolyte surveillance; persistent AKI should prompt differential expansion beyond prerenal disease |
| Sacituzumab govitecan / ATIN case [7] | Level D phenotype-defining case report | Metastatic ER-positive/PR-positive/HER2-negative breast cancer | SG exposure before AKI; case-level dosing and exposure reported in source | Single-patient report; no comparator | Temporal association with SG and clinical course reported | Severe AKI requiring hemodialysis; biopsy-proven acute tubulointerstitial nephritis; improved with corticosteroids | Vomiting and diarrhea co-occurred, creating potential prerenal confounding; proteinuria and biopsy clarified phenotype | Case-level creatinine course can support AKI interpretation, but incidence cannot be inferred | Expands the SG differential diagnosis to immune/interstitial injury when AKI is persistent or inflammatory |
| Mirvetuximab soravtansine / MIRASOL [8] | Level A randomized phase 3 trial | FRalpha-positive, platinum-resistant ovarian, fallopian tube, or primary peritoneal cancer | 6 mg/kg adjusted ideal body weight IV every 3 weeks | Investigator’s-choice chemotherapy; N=453 randomized (227 mirvetuximab; 226 chemotherapy) | Treatment until progression or unacceptable toxicity; trial population had 1-3 prior systemic lines | Renal injury was not presented as a dominant deeply phenotyped toxicity signal | Electrolyte outcomes were not standardized as tubular phenotype endpoints | Not KDIGO-mappable from available renal safety summaries alone | Improved global tolerability does not prove renal neutrality; mild/electrolyte-predominant injury may be under-ascertained |
| Mirvetuximab soravtansine / ELAHERE label [41] | Level B regulatory label | FRalpha-positive platinum-resistant ovarian cancer after 1-3 prior systemic regimens | 6 mg/kg adjusted ideal body weight IV every 3 weeks | Regulatory safety summaries from labeled development program | Exposure varies by treatment duration and dose modification | Kidney-specific adverse-event characterization remains limited compared with ocular and gastrointestinal toxicity domains | No standardized Fanconi-like or tubular biomarker endpoint set is provided in routine label summaries | Usually not KDIGO-stageable | Absence of a major creatinine signal should not be equated with absence of segment-selective renal injury |
| Trastuzumab deruxtecan / DESTINY-Breast03 [10] | Level A randomized phase 3 trial | HER2-positive unresectable or metastatic breast cancer previously treated with trastuzumab and taxane | T-DXd 5.4 mg/kg IV every 3 weeks | T-DM1 3.6 mg/kg IV every 3 weeks; N=524 randomized (261 T-DXd; 263 T-DM1) | Treatment until progression or unacceptable toxicity; subsequent updates report long follow-up | Renal adverse events were not presented as a deeply phenotyped endpoint in the pivotal comparison | Electrolyte clusters were not systematically reported as proximal tubule endpoints | Not generally KDIGO-mappable from main trial safety tables alone | Trial data support broad comparative safety context; proximal tubule hypothesis comes mainly from case-level evidence |
| Trastuzumab deruxtecan / ENHERTU label [38] | Level B regulatory label | Multiple HER2-expressing solid-tumor indications depending on label context | 5.4 mg/kg IV every 3 weeks for most solid-tumor settings; 6.4 mg/kg every 3 weeks in gastric cancer | Regulatory safety and pharmacokinetic summaries across labeled programs | Exposure varies by indication and dose level | Label highlights limited pharmacokinetic information in severe renal impairment | Routine label summaries do not provide a dedicated Fanconi/tubular endpoint panel | Not KDIGO-stageable from label terms alone | Supports caution in severe renal impairment and the need for prospective electrolyte/tubular monitoring |
| Trastuzumab deruxtecan / Fanconi case [9] | Level D phenotype-defining case report | Metastatic recurrent ER-positive/PR-negative/HER2-positive breast cancer | T-DXd exposure before electrolyte-wasting syndrome; case-level treatment course reported in source | Single-patient report; no comparator | Reversibility after drug withdrawal supports drug association | Fanconi-like proximal tubulopathy rather than generic renal impairment | Hypophosphatemia, hypokalemia, hypomagnesemia, non-anion-gap metabolic acidosis, normoglycemic glycosuria, and urinary phosphate/potassium wasting | Electrolyte cluster is not an AKI stage; creatinine course should be interpreted separately | High-specificity signal for proximal tubular phenotype; hypothesis-generating, not incidence-defining |

**Abbreviations:** ADC, antibody-drug conjugate; AKI, acute kidney injury; ATIN, acute tubulointerstitial nephritis; DKA, diabetic ketoacidosis; EV, enfortumab vedotin; FRalpha, folate receptor alpha; KDIGO, Kidney Disease: Improving Global Outcomes; SG, sacituzumab govitecan; T-DM1, trastuzumab emtansine; T-DXd, trastuzumab deruxtecan; TNBC, triple-negative breast cancer.
